# Accuracy of the Bronchoalveolar Lavage Enzyme-Linked Immunospot Assay for the Diagnosis of Pulmonary Tuberculosis: A Meta-analysis

**DOI:** 10.1097/MD.0000000000003183

**Published:** 2016-03-25

**Authors:** Caishuang Pang, Yanqiu Wu, Chun Wan, Konglong Shen, Yuzhu Hu, Ting Yang, Yongchun Shen, Fuqiang Wen

**Affiliations:** From the Department of Respiratory and Critical Care Medicine, West China Hospital of Sichuan University and Division of Pulmonary Diseases, State Key Laboratory of Biotherapy of China, Chengdu (CP, YW, CW, TY, YS, FW); Chongqing Cancer Hospital, Chongqing Cancer Institute, Chongqing (CP); Radiation Physics Center, Cancer Center and State Key Laboratory of Biotherapy, West China Hospital of Sichuan University (KS); and West China School of Medicine, Sichuan University, Chengdu, China (YH).

## Abstract

Assessing of local immune response may improve the accuracy of pulmonary tuberculosis (PTB) diagnosis. Many studies have investigated diagnosing PTB based on enzyme-linked immunospot (ELISPOT) assay of bronchoalveolar lavage (BAL) fluid, but the results have been inconclusive. We meta-analyzed the available evidences on overall diagnostic performance of ELISPOT assay of BAL fluid for diagnosing PTB.

A systematic literature search was performed using PubMed, Embase, Wangfang, Weipu, and CNKI. Data were pooled on sensitivity, specificity, positive likelihood ratio (PLR), negative likelihood ratio (NLR), and diagnostic odds ratio (DOR). Overall test performance was summarized using summary receiver operating characteristic curves and the area under the curve (AUC). Deeks test was used to test for potential publication bias.

Seven publications with 814 subjects met our inclusion criteria and were included in this meta-analysis. The following pooled estimates for diagnostic parameters were obtained: sensitivity, 0.90 (95% CI: 0.85–0.94); specificity, 0.80 (95% CI: 0.77–0.84); PLR, 5.08 (95% CI: 2.70–9.57); NLR, 0.13 (95% CI: 0.06–0.28); DOR, 49.12 (95% CI: 12.97–186.00); and AUC, 0.96. No publication bias was identified.

The available evidence suggests that ELISPOT assay of BAL fluid is a useful rapid diagnostic test for PTB. The results of this assay should be interpreted in parallel with clinical findings and the results of conventional tests.

## INTRODUCTION

Tuberculosis continues to be one of the world's biggest threats; it now ranks as a leading cause of infection-related mortality worldwide.^1,2^ In 2014, tuberculosis was diagnosed in an estimated 9.6 million people around the world, and it was linked to an estimated 1.5 million deaths.^1^ Pulmonary tuberculosis (PTB) is the major manifestation of the disease.^3^ Despite numerous diagnostic improvements, identification of *Mycobacterium tuberculosis* remains the gold standard for PTB diagnosis. However, culturing the pathogen is time-consuming, and false negative results are obtained for approximately 20% of patients with PTB.^4^

A rapid alternative is microscopic detection of acid-fast bacilli (AFB) in bronchial secretions or bronchoalveolar lavage (BAL) fluid. However, suitable specimens are difficult to obtain, sensitivity of microscopic detection has been shown to vary across 3 consecutive specimens,^5^ and the test gives false negative results in up to 50% of patients with PTB.^6^ Nucleic acid amplification tests (NAATs) can provide results several weeks earlier than culture, but these tests are often too expensive and complex for routine use in resource-limited settings. In addition, although NAATs show high specificity, their sensitivity can be variable and low.^7–10^ The automated, self-contained NAAT known as the Xpert MTB/RIF assay shows higher sensitivity and specificity than conventional NAATs^11,12^ for detecting *M. tuberculosis* and rifampicin resistance. However, it is expensive and available only in specialized institutions.^13,14^ The tuberculin skin test, while simple, can show false positive results in the presence of Bacille Calmette Guérin and many bacteria that are not *M. tuberculosis*, increasing the risk of misdiagnosis.^15^ The limitations of these diagnostic approaches highlight the need to identify new diagnostic tools.

Interferon-gamma release assays (IGRAs) are increasingly used to detect *M. tuberculosis* infection.^16^ IGRAs, known as the enzyme-linked immunospot (ELISPOT) assay and enzyme-linked immunosorbent assay (ELISA), are based on interferon-gamma (IFN-γ) secretion by lymphocytes exposed to *M. tuberculosis*-specific antigens: early secreted antigenic target 6 (ESAT-6) and culture filtrate protein 10 (CFP-10). Although IGRAs are now recommended as an alternative to the tuberculin skin test for targeted testing,^17,18^ the diagnostic accuracy of IGRAs show suboptimal diagnostic accuracy with both blood and pleural fluid samples.^19,20^ Studies suggest that applying IGRAs to BAL fluid may improve diagnostic performance, particularly for diagnosis of smear-negative PTB, since this fluid comes from the site of infection (lungs). After stimulation with ESAT-6 and CFP-10, the concentrations of T-cells secreting IFN-γ are 10- to 100-fold higher in BAL fluid than in peripheral blood in patient with PTB.^7,21^

Numerous studies have aimed to extend the use of BAL ELISPOT to the diagnosis of PTB, especially smear-negative PTB. The results have been variable, leading us to meta-analyze the available evidence to comprehensively assess the overall accuracy of BAL ELISPOT for diagnosing PTB.

## METHODS

### Search Strategy and Study Selection

A systematic search was performed using PubMed, Embase, Wanfang, Weipu, and CNKI to identify studies of the usefulness of BAL ELISPOT to diagnose PTB that were published up to July 2015. The following search terms were used: “bronchoalveolar lavage” OR “bronchoalveolar lavage fluid” AND “Enzyme-linked Immunospot” OR “ELISPOT” OR “Interferon-gamma release assays” OR “IGRA” AND “tuberculosis.” Articles were also identified using the “related-articles” function in PubMed. References within identified articles were also searched manually.

A study was included in this meta-analysis when it fulfilled the following inclusion criteria: it was an original research article published in English or Chinese; it applied the ELISPOT assay to BAL fluid for the diagnosis of PTB; it provided sufficient data about true positive, false positive, false negative, and true negative results; and it involved at least 20 participants to reduce selection bias. Institutional review board approval was not required for this retrospective meta-analysis.

### Data Extraction and Quality Assessment

Two reviewers independently assessed articles for eligibility to be included in this meta-analysis; discrepancies in assessment were resolved by consensus. The following data were retrieved from each study: 1st author, publication year, country, number of patients, diagnostic standard, test method, sample, numbers of true positive, numbers of false positive, numbers of false negative, numbers of true negative, indeterminate results, and methodological quality. The methodological quality of each study was assessed using the QUADAS-2 instrument.^22^ QUADAS-2 assesses risk of bias in 4 parts: patient selection, index test, reference standard, flow, and timing. It assesses applicability concerns in 3 parts: patient selection, index test, and reference standard.

### Statistical Analysis

Standard methods recommended for diagnostic accuracy meta-analysis were used.^23,24^ The following indices of test accuracy were computed for each study: sensitivity, specificity, positive likelihood ratio (PLR), negative likelihood ratio (NLR), diagnostic odds ratio (DOR), and the area under the curve (AUC). The analysis was based on a summary receiver operating characteristic (SROC) curve.^23,25^ Pooled estimates of sensitivity, specificity, and related indices were calculated across studies using a random-effects model or a fixed-effects model based on the results of heterogeneity tests. Heterogeneity between studies was evaluated using the χ^2^ test and Fisher exact tests. Deeks funnel plot was used to detect the potential presence of publication bias.^26^ Meta-analysis was carried out using STATA 12.0 (Stata Corp., College Station, TX), Meta-DiSc 1.4 (XI. Cochrane Colloquium, Barcelona, Spain), and RevMan 5.2 (Cochrane Collaboration, Oxford, UK). All statistical tests were 2-sided, and *P* < 0.05 was considered significant.

## RESULTS

### Characteristics and Quality of the Included Studies

**FIGURE 1:**
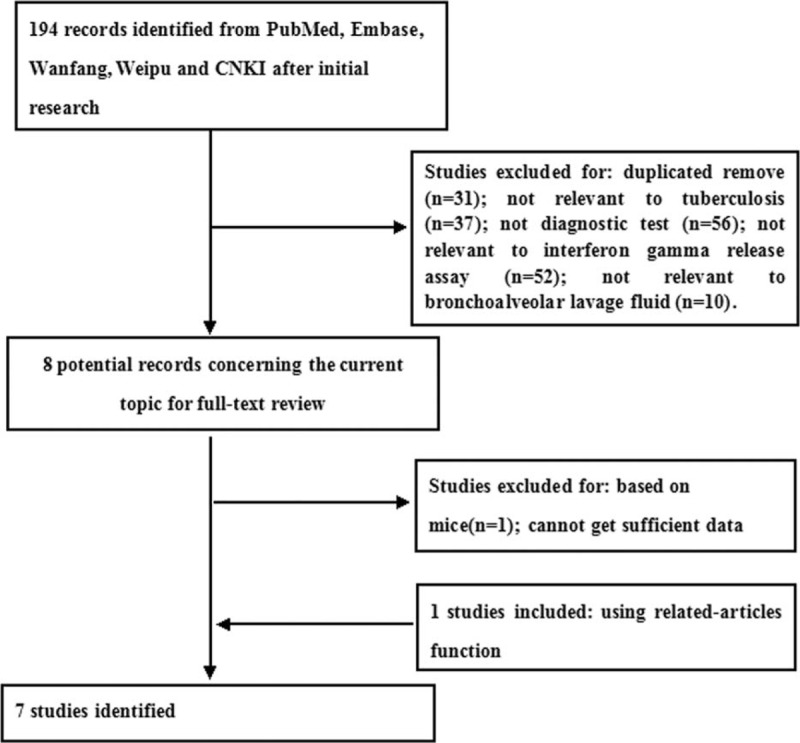
Flow diagram of included and excluded studies.

Seven studies assessing BAL ELISPOT for PTB diagnosis were included in the meta-analysis based on the inclusion criteria (Figure 1, Table 1).^27–33^ The average sample size was 116 (range, 32–347). All patients had been enrolled consecutively and prospectively. Patients were diagnosed with tuberculosis using a recently developed algorithm,^34^ and *M. tuberculosis* culture served as the reference standard. In all 7 studies, the ELISPOT-based T-SPOT-TB assay was performed. In 6 studies, the BAL ELISPOT result was considered positive when the test well contained at least 5 more spot-forming cells (SFCs) as the control well and when the test well contained twice the number of SFCs as the control well.^27–32^ The BAL ELISPOT result was considered negative if it did not meet the criteria for a positive result and if the positive control well contained at least twice the number of SFCs as the control well. Results that met neither of these definitions were considered to be indeterminate. In 1 study, in contrast, a BAL ELISPOT result was considered positive when the test well contained at least 6 more SFCs and had twice the number of SFCs as the control well.^33^

**TABLE 1:**
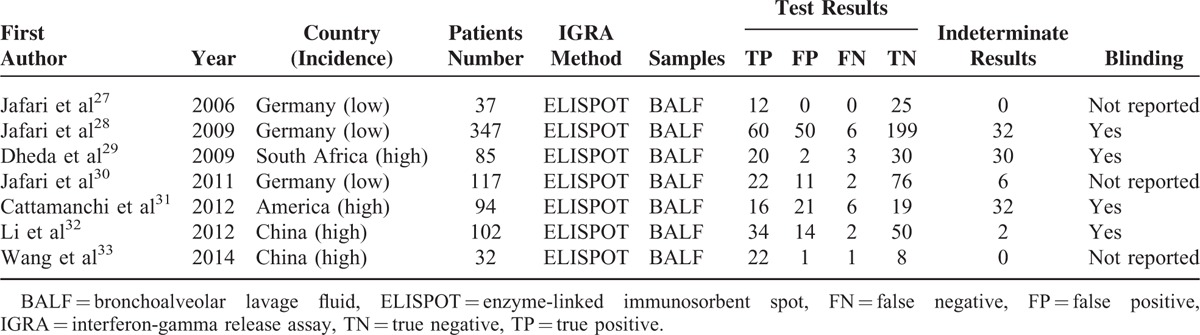
Clinical Summary of Included Studies Examining the Diagnostic Accuracy of Bronchoalveolar Lavage Enzyme-Linked Immunospot

**FIGURE 2:**
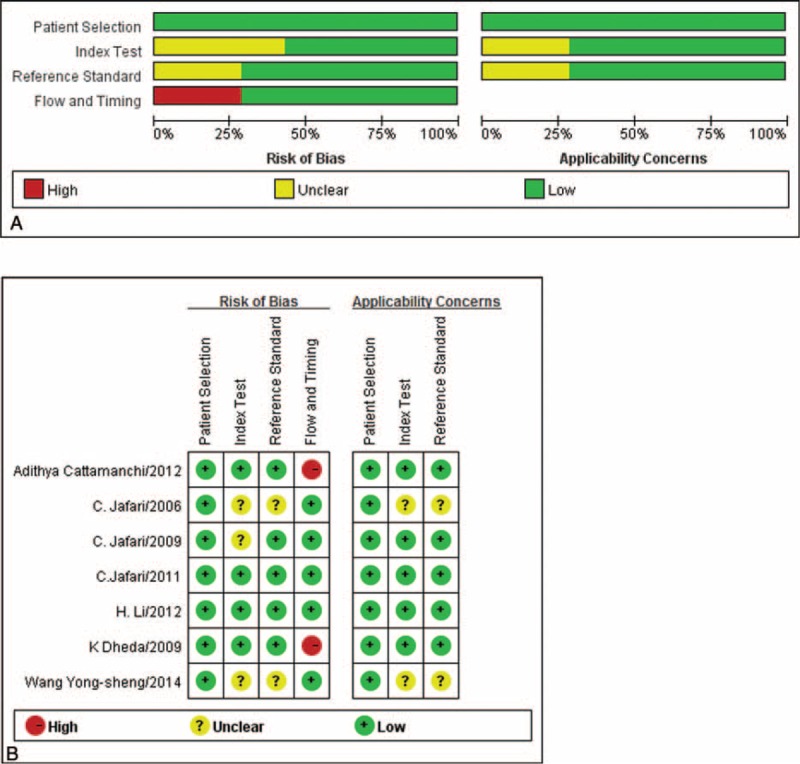
Quality assessment of studies of the bronchoalveolar lavage enzyme-linked immunospot assay. (A) Graph of risk of bias and applicability concerns. (B) Summary of risk of bias and applicability concerns.

QUADAS-2 was proposed in 2011 and was integrated into RevMan 5.2 in 2012.^22,35^ This instrument assesses methodological quality in terms of patient selection, index test, reference standard, and flow and timing. When a criterion is fulfilled, an answer of Yes is given; if it is unclear whether a criterion is fulfilled, Unclear is reported; and if a criterion is not fulfilled, No is reported. These responses for each criterion are then converted into risk of bias and applicability concerns. The quality of studies in our meta-analysis was generally good, but 3 studies were judged to have unclear risk of bias (Figure 2). This risk of bias was related to the index test in all 3 studies^27,28,33^ as well as to the reference standard in 2 of them.^27,33^ Two studies were judged to have high risk of bias,^29,31^ related to flow and timing.

### Diagnostic Accuracy

**FIGURE 3:**
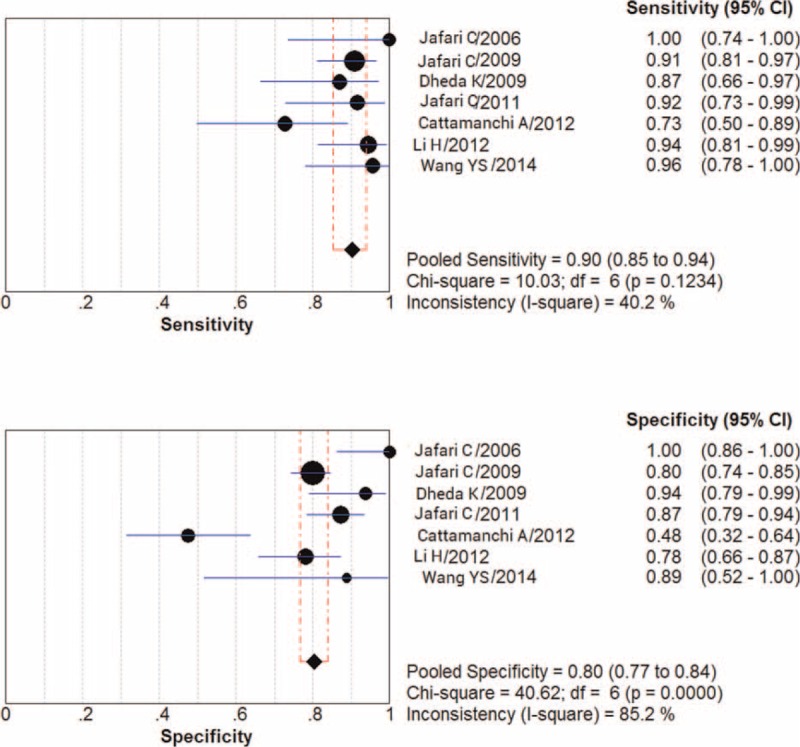
Forest plot showing estimates of sensitivity and specificity for the bronchoalveolar lavage enzyme-linked immunospot assay. Point estimates of sensitivity and specificity from each study are shown as solid circles. Error bars indicate 95% confidence interval (CI).

**FIGURE 4:**
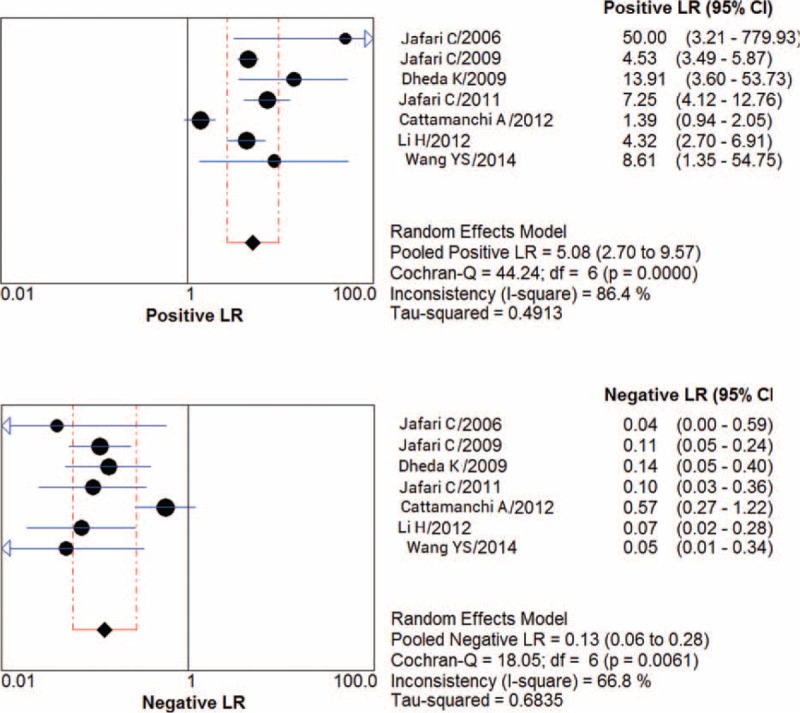
PLR and NLR for the bronchoalveolar lavage enzyme-linked immunospot assay. NLR = negative likelihood ratio, PLR = positive likelihood ratio.

Figure 3 shows estimates of sensitivity and specificity for diagnostic accuracy of BAL ELISPOT. Sensitivity ranged from 0.73 to 1.00 (pooled: 0.90; 95% CI: 0.85–0.94), and specificity ranged from 0.48 to 1.00 (pooled: 0.80; 95% CI: 0.77–0.84). The following pooled parameters were also calculated: PLR, 5.08 (95% CI: 2.70–9.57); NLR, 0.13 (95% CI: 0.06–0.28) (Figure 4); and DOR, 49.12 (95% CI: 12.97–186.00). Chi-squared values for these pooled estimates were 10.03 (*P* = 0.123) for sensitivity, 40.62 (*P* = 0.000) for specificity, 44.24 (*P* = 0.000) for PLR, 18.05 (*P* = 0.006) for NLR, and 28.21 (*P* = 0.000) for DOR. These values indicate significant heterogeneity among the studies.

In the subset of 3 studies conducted in settings with low tuberculosis incidence,^27,28,30^ pooled sensitivity was 0.92 (95% CI: 0.85–0.97) and specificity was 0.83 (95% CI: 0.79–0.87). Among the remaining studies, conducted in settings with high tuberculosis incidence,^29,31–33^ pooled sensitivity was 0.88 (95% CI: 0.81–0.94) and specificity was 0.74 (95% CI: 0.66–0.81). Comparing these subgroup analyses with results pooled from all 7 studies suggests that BAL ELISPOT performs better in settings of low tuberculosis incidence, but the difference in performance in the 3 cases was not statistically significant (*P* = 0.585).

**FIGURE 5:**
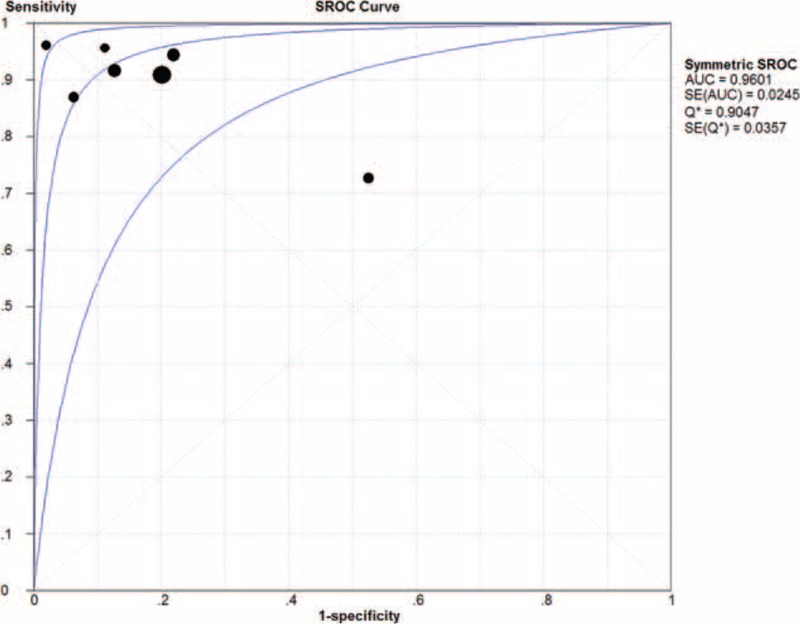
Summary receiver operating characteristic (SROC) curves for the bronchoalveolar lavage enzyme-linked immunospot assay. Solid circles represent each study included in the meta-analysis. The size of each study is indicated by the size of the solid circle. The regression SROC curves summarize the overall diagnostic accuracy.

An SROC curve was calculated for BAL ELISPOT, this curve plots sensitivity against 1-specificity from individual studies (Figure 5). As a global measure of test efficacy, we determined the Q-value; this is the point of intersection between the SROC curve and the diagonal running from the left upper corner to the lower right corner of the ROC space. The Q-value corresponds to the highest common value of sensitivity and specificity, as well as the point where sensitivity equals specificity. The SROC curve was positioned near the desirable upper left corner of the plot, suggesting good performance, and Q-value for sensitivity and specificity was 0.90 (SEM 0.036), with an AUC of 0.96 (SEM 0.025). These data indicated high overall accuracy for the BAL ELISPOT assay in PTB diagnosis.

### Multiple Regression Analysis and Publication Bias

The effect of study quality on the relative DOR of BAL ELISPOT was assessed by performing meta-aggression involving setting (low vs high tuberculosis incidence), sample size, blinding design, publication year, and rate of indeterminate results (Table 2). Three studies were performed in areas with a low tuberculosis incidence^27,28,30^ and 4 studies in areas with high tuberculosis incidence.^29,31–33^ Two studies showed rates of indeterminate results >30%.^29,31^ Meta-regression showed that setting, sample size, blinding design, and publication year did not substantially affect the diagnostic accuracy of BAL ELISPOT (*P* > 0.05). However, indeterminate results did affect diagnostic accuracy (*P* = 0.013). In addition, we found that the inclusion of HIV patients in 2 studies^29,31^ significantly affected heterogeneity (*P* = 0.013).

**TABLE 2:**
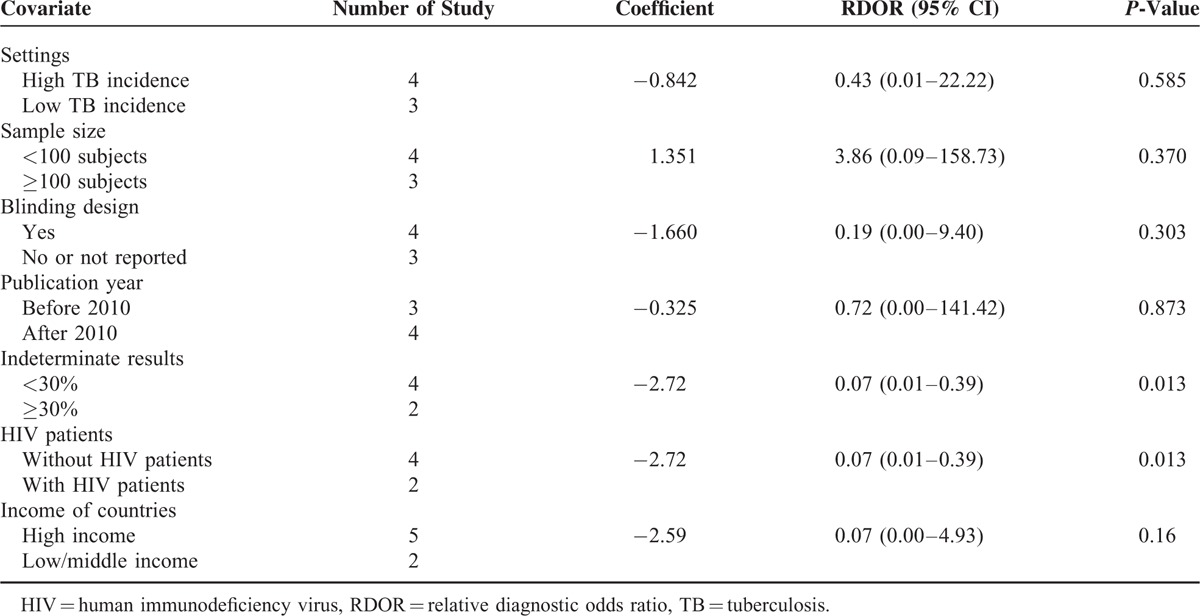
Meta-Regression on Diagnostic Accuracy of Bronchoalveolar Lavage Enzyme-Linked Immunospot

**FIGURE 6:**
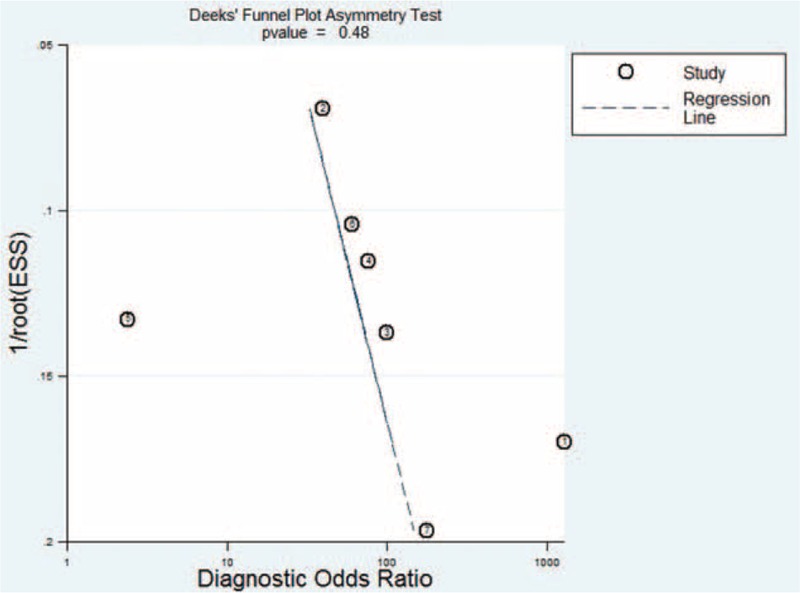
Funnel graph to assess risk of publication bias in studies of the bronchoalveolar lavage enzyme-linked immunospot assay. The funnel graph plots the log of the diagnostic odds ratio (DOR) against the standard error of the log of the DOR (an indicator of sample size). Solid circles represent each study in the meta-analysis. The regression line is shown.

Deeks funnel plot asymmetry test was used to assess likelihood of publication bias. Although the funnel plots for publication bias showed some asymmetry due to the limited number of studies (Figure 6), the *P* value associated with Deeks test was not significant (*P* = 0.48). This suggests symmetry in the data and low likelihood of publication bias.

## DISCUSSION

Currently, 2 types of IGRAs are commercially available: the ELISA-based QFT-G or QFT-IT, and the ELISPOT-based T-SPOT-TB. Both ELISPOT and ELISA measure IFN-γ release after T cell stimulation with the *M. tuberculosis*-specific antigens ESAT-6 and CFP-10. However, ELISPOT has been reported to be more stable and sensitive.^15,36^ This has led investigators to examine whether ELISPOT may be useful for tuberculosis screening. Those studies have focused on BAL fluid, since IGRAs show inadequate sensitivity and specificity with pleural and blood samples.^19,20,24^ Assaying BAL fluid may boost sensitivity since *M. tuberculosis*-specific lymphocytes are concentrated at the site of infection (lungs).^27^ Given the rationality of local immunodiagnosis using BAL ELISPOT,^37,38^ a growing number of studies have evaluated the diagnostic accuracy of BAL ELISPOT for PTB, but results have been mixed.

Our meta-analysis of the available evidence showed a pooled sensitivity of 0.90 and specificity of 0.80. The relatively high sensitivity indicates that it would be possible to exclude PTB if a patient's BAL ELISPOT result was below the cut-off value. However, the moderate specificity limits the usefulness of the test. At the same time, our meta-analysis indicated an AUC of 0.96 for the SROC curve, which presents a global summary of test performance and shows the trade-off between sensitivity and specificity. Since an AUC of 1.0 (100%) indicates perfect ability to discriminate cases from noncases, our meta-analysis suggests a relatively high level of overall diagnostic accuracy. Consistent with this, we calculated a pooled DOR of 49.12 for BAL ELISPOT. The DOR indicates the ratio of the odds of positive test results in the diseased group to the odds of positive test results in the nondiseased group. Higher values of DOR, which ranges from 0 to infinity, indicate better discriminatory performance. The pooled DOR in our meta-analysis suggests that the assay is helpful for diagnosing PTB. Our results are consistent with reports that BAL ELISPOT is a more sensitive supplementary test than blood or pleural ELISPOT.^36^ In addition, BAL fluid is easy to obtain in patients who are unable to produce sputum. Thus, BAL ELISPOT may afford an alternative for patients without sputum.

Since the PLR and NLR are more clinically meaningful and easier to interpret than the AUC, we used these indices to assess the diagnostic accuracy of BAL ELISPOT. PLR indicates how much the odds of a condition are increased by a positive test, while NLR indicates how much they are decreased by a negative test. Larger PLR means greater diagnostic accuracy, whereas a smaller NLR is better. Our meta-analysis indicated a pooled PLR of 5.08, meaning that patients with PTB had a 5-fold higher chance of giving a positive BAL ELISPOT result than did patients without PTB. This is not a large enough difference for clinical use. Similarly, the pooled NLR was 0.13, indicating 13% probability that a patient with a negative BAL ELISPOT result actually had tuberculosis, which is not low enough to exclude PTB in the clinic.

Based on World Bank criteria, 5 studies in our meta-analysis were conducted in high-income countries,^27–31^ while 2 were conducted in low- or middle-income countries.^32,33^ Five of 7 studies used the tuberculin skin test, which gave results consistent with BAL ELISPOT but with lower sensitivity and specificity.^27–30,32^ A recent meta-analysis concluded that the overall sensitivity of the tuberculin skin test for active TB is 77%,^39^ which is lower than the 90% calculated here for BAL ELISPOT. In addition, the tuberculin skin test is influenced by previous tuberculosis history, while BAL ELISPOT is not. However, BAL ELISPOT is not available in resource-limited settings, where the budget and infrastructure for IGRAs are lacking. This helps to explain why, even though IGRAs have been available for several years, the tuberculin skin test is still much more widely used to screen people with a positive immune response against *M. tuberculosis*.^40^ Studies suggest that if an individual gives negative results on both the tuberculin skin test and an IGRA, active tuberculosis can be ruled out with greater than 95% confidence.^41,42^

The gold standard for tuberculosis diagnosis remains culture of *M. tuberculosis* in liquid or solid media. Both types of culture require weeks, and this assay fails to detect approximately 20% of PTB cases.^4^ The pathogen can be detected microscopically in an AFB assay, which is rapid but of limited sensitivity.^6^ Although an *M. tuberculosis*-specific NAAT is frequently used for rapid PTB diagnosis, the sensitivity of NAAT with sputum or BAL fluid is good in patients with positive results on AFB sputum smears,^43^ but it gives false negative results for more than 50% of patients with negative smear results.^8,44^ Similarly, the Xpert MTB/RIF assay, an automated NAAT that performs much better in patients with PTB than conventional NAATs,^11,12^ shows higher sensitivity in smear-positive patients (98%) than in smear-negative ones (67%).^13^ Thus, studies have concluded that although the Xpert assay can diagnose PTB better than smear microscopy, its overall sensitivity is less than that of the culture test.^45–47^ In addition, Xpert MTB/RIF requires a stable and uninterrupted electrical power supply, temperature control, and annual calibration of the cartridge modules.^14^ These considerations indicate that no existing microbiological or immunological method on its own is adequate to diagnose or exclude PTB.

Since guidelines recommend bronchoscopy for patients with suspected tuberculosis who give negative results on AFB sputum smears,^30,34^ we suggest that a negative smear result should lead to prompt bronchoscopy and BAL ELISPOT. Indeed, a comparison of various methods to diagnose active PTB (BAL fluid microscopy, BAL fluid culture, transbronchial biopsy for histology, BAL NAAT, and BAL ELISPOT) concluded that BAL ELISPOT was the best method for rapid diagnosis.^30^

Two of 7 studies included in this meta-analysis used BAL NAAT for the diagnosis of PTB.^28,30^ The percentage of patients with PTB not diagnosed by NAAT was 64.8%, compared to only 14.1% for BAL ELISPOT.^28^ The BAL NAAT showed lower sensitivity and specificity than BAL ELISPOT. These results suggest that BAL ELISPOT shows superior, more consistent diagnostic performance than BAL NAAT, especially for patients with smear-negative PTB. Thus, BAL ELISPOT provides an alternative technique for detecting PTB associated with negative results in the AFB smear test and culture test. At the same time, the expense, invasiveness and risk of obtaining BAL fluid, and assaying it means that this alternative may not be appropriate for all patients in all settings.

Five of 7 studies included in this meta-analysis also examined blood ELISPOT for PTB diagnosis.^27–31^ The diagnostic accuracy of BAL-ELISPOT is superior to that of blood ELISPOT, probably reflecting the fact that in active PTB, antigen-specific T cells clonally expand and concentrate at the site of infection (lungs). In addition, a positive BAL ELISPOT result effectively discriminates between active and latent tuberculosis.^28–30^

Significant heterogeneity was detected among the included studies, so we performed meta-regression analysis to investigate the possible sources of this heterogeneity. Although diagnostic accuracy of BAL ELISPOT tended to be higher in settings of low tuberculosis incidence than in settings of high incidence, the difference was not significant. In contrast, the relatively high rate of indeterminate results did affect the diagnostic accuracy of BAL ELISPOT (*P* = 0.013), as did inclusion of HIV patients^29,31^ (*P* = 0.013). These results are consistent with reports that the large proportion of indeterminate results makes the BAL ELISPOT less than ideal in areas of high tuberculosis incidence where immunodiagnosis focuses on the lung.^29,31^ They are also consistent with a report that BAL ELISPOT performed poorly in an area with high HIV burden.^31^ The presence of immunosuppressive cytokines or cells, including immunosuppressive pulmonary macrophages and regulatory T cells, may help explain the high proportion of inconclusive results.^48^ Another contributor may be high spot count in the negative control.^29^ A 3rd reason may be that the IFN-γ detected in the assay is secreted by terminally differentiated effector T-cells rather than by *M. tuberculosis*-specific effector T-cells. The accumulation of terminally differentiated effector T-cells is a hallmark of early immune senescence in advanced HIV/AIDS in South Africa,^31,49^ and terminally differentiated effector T-cells may concentrate at the site of disease in patients with active PTB.^50^ The 4th reason may be low numbers of cells harvested at bronchoscopy, particularly in technically difficult procedures. Multicenter clinical studies should be performed to rigorously assess the diagnostic performance of BAL ELISPOT in patients with PTB.

We used the QUADAS-2 instrument^22^ to assess the quality of included studies on the basis of information in the title, Introduction, Methods, Results, and Discussion. This is an improved version of the original QUADAS instrument^51^ that takes into account more details of the study, such as the explanation of indeterminate results. Since the studies often reported inadequate information, assessment of several criteria was “unclear,” increasing risk of bias and applicability concerns.

Our meta-analysis is limited by several factors. First, only 7 publications were included, reflecting the strict search strategy and study selection. The limited patient numbers may have influenced the outcomes, so statistical power may be inadequate for drawing definitive conclusions about the ability of BAL ELISPOT to diagnose PTB. Further, larger studies may be needed to confirm the diagnostic value of BAL ELISPOT. Second, because of the few studies included, we were unable to perform subgroup analysis to assess studies that included HIV patients separately from other studies. Further study of BAL ELISPOT for diagnosing PTB in HIV patients is needed, which will allow rigorous meta-analysis to be carried out. Third, all the studies in our meta-analysis were of modest methodological quality, based on QUADAS-2 assessment. This was due mainly to lack of reporting of key information.

## CONCLUSIONS

The present meta-analysis suggests that BAL ELISPOT may significantly aid the diagnosis of PTB. Its relatively high sensitivity may make it suitable for PTB screening. Further study and optimization are required to reduce the rate of inconclusive results. Although BAL ELISPOT shows better diagnostic performance than both the tuberculin skin test and blood ELISPOT, it is not accurate enough on its own to diagnose or exclude PTB. Effective combination of different diagnostic tests remains the most critical element in controlling tuberculosis.

## References

[1] World Health Organization. *Global Tuberculosis Report: WHO Report 2015.* WHO/HTM/TB/2015.22.

[2] Giacomini G Miranda JR Pavan AL Quantification of pulmonary inflammatory processes using chest radiography: tuberculosis as the motivating application. *Medicine* 2015; 94:e1044. 26131814 10.1097/MD.0000000000001044 PMC4504622

[3] Dye C Scheele S Dolin P Consensus statement. Global burden of tuberculosis: estimated incidence, prevalence, and mortality by country. WHO global surveillance and monitoring project. *JAMA* 1999; 282:677–686. 10517722 10.1001/jama.282.7.677

[4] Strassburg A Jafari C Ernst M Rapid diagnosis of pulmonary TB by BAL enzyme-linked immunospot assay in an immunocompromised host. *Eur Respir J* 2008; 31:1132–1135. 18448508 10.1183/09031936.00083707

[5] American Thoracic Society Centers for Disease Control and Prevention. Diagnostic standards and classification of tuberculosis in adults and children. *Am J Respir Crit Care Med* 2000; 161:1376–1395. 10764337 10.1164/ajrccm.161.4.16141

[6] Daniel TM The rapid diagnosis of tuberculosis: a selective review. *J Lab Clin Med* 1990; 116:277–282. 1698213

[7] Barry SM Lipman MC Bannister B Purified protein derivative-activated type 1 cytokine-producing CD4+ T lymphocytes in the lung: a characteristic feature of active pulmonary and nonpulmonary tuberculosis. *J Infect Dis* 2003; 187:243–250. 12552448 10.1086/346112

[8] Sarmiento OL Weigle KA Alexander J Assessment by meta-analysis of PCR for diagnosis of smear-negative pulmonary tuberculosis. *J Clin Microbiol* 2003; 41:3233–3240. 12843069 10.1128/JCM.41.7.3233-3240.2003 PMC165327

[9] Greco S Girardi E Navarra A Current evidence on diagnostic accuracy of commercially based nucleic acid amplification tests for the diagnosis of pulmonary tuberculosis. *Thorax* 2006; 61:783–890. 16738037 10.1136/thx.2005.054908 PMC2117107

[10] Ling DI Flores LL Riley LW Commercial nucleic-acid amplification tests for diagnosis of pulmonary tuberculosis in respiratory specimens: meta-analysis and meta-regression. *PLoS One* 2008; 3:e1536. 18253484 10.1371/journal.pone.0001536 PMC2212137

[11] Khalil KF Butt T Diagnostic yield of bronchoalveolar lavage gene Xpert in smear-negative and sputum-scarce pulmonary tuberculosis. *J Coll Physicians Surg Pak* 2015; 25:115–118. 25703755

[12] Ko Y Lee HK Lee YS Accuracy of Xpert(®) MTB/RIF assay compared with AdvanSure™ TB/NTM real-time PCR using bronchoscopy specimens. *Int J Tuberc Lung Dis* 2016; 20:115–120. 26688537 10.5588/ijtld.15.0227

[13] Steingart KR Schiller I Horne DJ Xpert® MTB/RIF assay for pulmonary tuberculosis and rifampicin resistance in adults. *Cochrane Database Syst Rev* 2014; (Issue 1): [DOI:10.1002/14651858.CD009593. pub3]. 10.1002/14651858.CD009593.pub3 PMC4470349 24448973

[14] World Health Organization. Rapid Implementation of the Xpert MTB/RIF Diagnostic Test. Technical and Operational ‘How-to’. Practical Considerations. WHO/HTM/TB/2011. 2. World Health Organization: Geneva; 2011.

[15] Liebeschuetz S Bamber S Ewer K Diagnosis of tuberculosis in South African children with a T-cell-based assay: a prospective cohort study. *Lancet* 2004; 364:2196–2203. 15610806 10.1016/S0140-6736(04)17592-2

[16] Dheda K Udwadia ZF Huggett JF Utility of the antigen-specific interferon-gamma assay for the management of tuberculosis. *Curr Opin Pulm Med* 2005; 11:195–202. 15818179 10.1097/01.mcp.0000158726.13159.5e

[17] Mazurek GH Jereb J Vernon A Updated guidelines for using interferon gamma release assays to detect mycobacterium tuberculosis infection-United States. *MMWR Recomm Rep* 2010; 59:1–25. 20577159

[18] Canadian Tuberculosis Committee. Updated recommendations on interferon gamma release assays for latent tuberculosis infection. *Can Commun Dis Rep* 2010; 36:1–21. 18979589

[19] Metcalfe JZ Everett CK Steingart KR Interferon-gamma release assays for active pulmonary tuberculosis diagnosis in adults in low- and middle-income countries: systematic review and meta-analysis. *J Infect Dis* 2011; 204 suppl 4:1120–1129. 21996694 10.1093/infdis/jir410 PMC3192542

[20] Cattamanchi A Smith R Steingart KR Interferon-gamma release assays for the diagnosis of latent tuberculosis infection in HIV-infected individuals: a systematic review and meta-analysis. *J Acquir Immune Defic Syndr* 2011; 56:230–238. 21239993 10.1097/QAI.0b013e31820b07ab PMC3383328

[21] Barnes PF Lu S Abrams JS Cytokine production at the site of disease in human tuberculosis. *Infect Immun* 1993; 61:3482–3489. 8335379 10.1128/iai.61.8.3482-3489.1993 PMC281026

[22] Whiting PF Rutjes AW Westwood ME QUADAS- 2: a revised tool for the quality assessment of diagnostic accuracy studies. *Ann Intern Med* 2011; 155:529–536. 22007046 10.7326/0003-4819-155-8-201110180-00009

[23] Devillé WL Buntinx F Bouter LM Conducting systematic reviews of diagnostic studies: didactic guidelines. *BMC Med Res Methodol* 2002; 2:9. 12097142 10.1186/1471-2288-2-9 PMC117243

[24] Pang CS Shen YC Tian PW Accuracy of the interferon-gamma release assay for the diagnosis of tuberculous pleurisy: an updated meta-analysis. *PeerJ* 2005; 3:e951. 26038718 10.7717/peerj.951 PMC4451019

[25] Moses LE Shapiro D Littenberg B Combining independent studies of a diagnostic test into a summary ROC curve: data-analytic approaches and some additional considerations. *Stat Med* 1993; 12:1293–1316. 8210827 10.1002/sim.4780121403

[26] Deeks JJ Macaskill P Irwig L The performance of tests of publication bias and other sample size effects in systematic reviews of diagnostic test accuracy was assessed. *J Clin Epidemiol* 2005; 58:882–893. 16085191 10.1016/j.jclinepi.2005.01.016

[27] Jafari C Ernst M Kalsdorf B Rapid diagnosis of smear-negative tuberculosis by bronchoalveolar lavage enzyme-linked immunospot. *Am J Respir Crit Care Med* 2006; 174:1048–1054. 16858013 10.1164/rccm.200604-465OC

[28] Jafari C Thijsen S Sotgiu G Bronchoalveolar lavage enzyme-linked immunospot for a rapid diagnosis of tuberculosis. *Am J Respir Crit Care Med* 2009; 180:666–673. 19590020 10.1164/rccm.200904-0557OC PMC2753791

[29] Dheda K van Zyl-Smit K Meldau R Quantitative lung T cell responses aid the rapid diagnosis of pulmonary tuberculosis. *Thorax* 2009; 64:847–853. 19592392 10.1136/thx.2009.116376

[30] Jafari C Kessler P Sotgiu G Impact of a Mycobacterium tuberculosis-specific interferon-(release assay in bronchoalveolar lavage fluid for a rapid diagnosis of tuberculosis. *J Intern Med* 2011; 270:254–262. 21418341 10.1111/j.1365-2796.2011.02378.x

[31] Cattamanchi A Sewenyana I Nabatanzi R Bronchoalveolar lavage enzyme-linked immunospot for diagnosis of smear-negative tuberculosis in HIV-infected patients. *PLoS One* 2012; 7:e39838. 22745833 10.1371/journal.pone.0039838 PMC3383728

[32] Li H Yang L Zheng CY Use of bronchoalveolar lavage enzyme-linked immunospot for diagnosis of smear-negative pulmonary tuberculosis. *Int J Tuberc Lung Dis* 2012; 16:1668–1673. 23131267 10.5588/ijtld.12.0292

[33] Wang YS Ning L Jiang FP The application value of TSPOT. TB in bronchoalveolar lavage fluid to diagnose active tuberculosis. *Sichuan Med J* 2014; 35:423–424.

[34] Lange C Mori T Advances in the diagnosis of tuberculosis. *Respirology* 2010; 15:220–240. 20199641 10.1111/j.1440-1843.2009.01692.x

[35] Review Manager (RevMan) [Computer program] Version 5.2. Copenhagen: The Nordic Cochrane Centre, The Cochrane Collaboration, 2012.

[36] Zhou Q Chen YQ Qin SM Diagnostic accuracy of T-cell interferon-γ release assays in tuberculous pleurisy: a meta-analysis. *Respirology* 2011; 16:473–480. 21299686 10.1111/j.1440-1843.2011.01941.x

[37] Wilkinson KA Wilkinson RJ Pathan A Ex vivo characterization of early secretory antigenic target 6-specific T cells at sites of active disease in pleural tuberculosis. *Clin Infect Dis* 2005; 40:184–187. 15614710 10.1086/426139

[38] Jafari C Ernst M Strassburg A Local immunodiagnosis of pulmonary tuberculosis by enzyme-linked immunospot. *Eur Respir J* 2008; 31:261–265. 17989118 10.1183/09031936.00096707

[39] Pai M Zwerling A Menzies D Systematic review: T-cell-based assays for the diagnosis of latent tuberculosis infection: an update. *Ann Intern Med* 2008; 149:177–184. 18593687 10.7326/0003-4819-149-3-200808050-00241 PMC2951987

[40] Bothamley GH Ditiu L Migliori GB Active case finding of tuberculosis in Europe: a tuberculosis network European trials group (TBNET) survey. *Eur Respir J* 2008; 32:1023–1030. 18550615 10.1183/09031936.00011708

[41] Dosanjh DP Hinks TS Innes JA Improved diagnostic evaluation of suspected tuberculosis. *Ann Intern Med* 2008; 148:325–336. 18316751 10.7326/0003-4819-148-5-200803040-00003 PMC2761734

[42] Goletti D Stefania C Butera O Accuracy of immunodiagnostic tests for active tuberculosis using single and combined results: a multicenter TBNET-Study. *PLoS One* 2008; 3:e3417. 18923709 10.1371/journal.pone.0003417 PMC2561073

[43] Greco S Rulli M Girardi E Diagnostic accuracy of in-house PCR for pulmonary tuberculosis in smear-positive patients: meta-analysis and meta-regression. *J Clin Microbiol* 2009; 47:569–576. 19144797 10.1128/JCM.02051-08 PMC2650956

[44] Hillemann D Weizenegger M Kubica T Use of the geno-Type MTB DR assay for rapid detection of rifampin and isoniazid resistance in Mycobacterium tuberculosis complex isolates. *J Clin Microbiol* 2005; 43:3699–3703. 16081898 10.1128/JCM.43.8.3699-3703.2005 PMC1233903

[45] World Health Organization. Policy Statement: Automated Real-Time Nucleic Acid Amplification Technology for Rapid and Simultaneous Detection of Tuberculosis and Rifampicin Resistance: Xpert MTB/RIF System. WHO/HTM/TB/2011. 4. Geneva: World Health Organization; 2011. 26158191

[46] World Health Organization, World Health Organization. Policy Update: Xpert MTB/RIF Assay for the Diagnosis of Pulmonary and Extrapulmonary TB in Adults and Children, 2013. 2013. 25473701

[47] Detjen AK DiNardo AR Leyden J Xpert MTB/RIF assay for the diagnosis of pulmonary tuberculosis in children: a systematic review and meta-analysis. *Lancet Respir Med* 2015; 3:451–461. 25812968 10.1016/S2213-2600(15)00095-8 PMC4756280

[48] Guyot-Revol V Innes JA Hackforth S Regulatory T cells are expanded in blood and disease sites in tuberculosis patients. *Am J Respir Crit Care Med* 2006; 173:803–810. 16339919 10.1164/rccm.200508-1294OC

[49] Desai S Landay A Early immune senescence in HIV disease. *Curr HIV/AIDS Rep* 2010; 7:4–10. 20425052 10.1007/s11904-009-0038-4 PMC3739442

[50] Caccamo N Meraviglia S La Mendola C Phenotypical and functional analysis of memory and effector human CD8 T cells specific for mycobacterial antigens. *J Immunol* 2006; 177:1780–1785. 16849488 10.4049/jimmunol.177.3.1780

[51] Whiting P Rutjes AW Reitsma JB The development of QUADAS: a tool for the quality assessment of studies of diagnostic accuracy included in systematic reviews. *BMC Med Res Methodol* 2003; 3:25. 14606960 10.1186/1471-2288-3-25 PMC305345

